# Predicting the Need for Therapeutic Intervention and Mortality in Acute Pancreatitis: A Two-Center International Study Using Machine Learning

**DOI:** 10.3390/jpm12040616

**Published:** 2022-04-11

**Authors:** Na Shi, Lan Lan, Jiawei Luo, Ping Zhu, Thomas R. W. Ward, Peter Szatmary, Robert Sutton, Wei Huang, John A. Windsor, Xiaobo Zhou, Qing Xia

**Affiliations:** 1 Department of Integrated Traditional Chinese and Western Medicine, Sichuan Provincial Pancreatitis Centre and West China-Liverpool Biomedical Research Centre, West China Hospital, Sichuan University, Chengdu 610044, China; nashi@scu.edu.cn (N.S.); zhuping@wchscu.cn (P.Z.); dr_wei_huang@scu.edu.cn (W.H.); 2 IT Center, Beijing Tiantan Hospital, Capital Medical University, Beijing 100070, China; 3 West China Biomedical Big Data Centre, West China Hospital, Sichuan University, Chengdu 610041, China; luojiawei@wchscu.cn; 4 Liverpool Pancreatitis Study Group, Royal Liverpool University Hospital, Institute of Translational Medicine, University of Liverpool, Liverpool L6 93BX, UK; thomasrwward@doctors.org.uk (T.R.W.W.); pszatmary@me.com (P.S.); r.sutton@liverpool.ac.uk (R.S.); 5 Surgical and Translational Research Centre, Faculty of Medical and Health Sciences, University of Auckland, Auckland 1023, New Zealand; j.windsor@auckland.ac.nz; 6 School of Biomedical Informatics, University of Texas Health Science Centre at Houston, Houston, TX 77030, USA; xiaobo.zhou@uth.tmc.edu

**Keywords:** acute pancreatitis, machine learning, predictor, interventions, mortality

## Abstract

Background: Current approaches to predicting intervention needs and mortality have reached 65–85% accuracy, which falls below clinical decision-making requirements in patients with acute pancreatitis (AP). We aimed to accurately predict therapeutic intervention needs and mortality on admission, in AP patients, using machine learning (ML). Methods: Data were obtained from three databases of patients admitted with AP: one retrospective (Chengdu) and two prospective (Liverpool and Chengdu) databases. Intervention and mortality differences, as well as potential predictors, were investigated. Univariate analysis was conducted, followed by a random forest ML algorithm used in multivariate analysis, to identify predictors. The ML performance matrix was applied to evaluate the model’s performance. Results: Three datasets of 2846 patients included 25 potential clinical predictors in the univariate analysis. The top ten identified predictors were obtained by ML models, for predicting interventions and mortality, from the training dataset. The prediction of interventions includes death in non-intervention patients, validated with high accuracy (96%/98%), the area under the receiver-operating-characteristic curve (0.90/0.98), and positive likelihood ratios (22.3/69.8), respectively. The post-test probabilities in the test set were 55.4% and 71.6%, respectively, which were considerably superior to existing prognostic scores. The ML model, for predicting mortality in intervention patients, performed better or equally with prognostic scores. Conclusions: ML, using admission clinical predictors, can accurately predict therapeutic interventions and mortality in patients with AP.

## 1. Introduction

Acute pancreatitis (AP) is one of the most common admission diagnoses relating to an acute gastrointestinal pathology. Approximately 25% of patients with AP develop infected pancreatic necrosis (IPN) and/or organ failure (OF), with mortality rates of 20–50% [1,2]. While the outcome of patients with AP has improved over recent decades, AP incidence and associated disability remain high [3], and specific drug therapies remain unavailable [4]. One of the challenges of therapeutic trials for AP is the inaccuracy in early severity and complication prediction, resulting in heterogeneous treatment groups. A review of current predictors of AP outcome [5] (including IPN, OF, and the need for intervention) demonstrated that the accuracy of current systems ranges from 65% to 85%, implying a misclassification error of 15–35%. This degree of inaccuracy in the prediction has clinical and research practice consequences.

Improving the accuracy of early severity prediction is of paramount importance and a matter of significant international effort. Various individual serum biomarkers have been investigated. However, they have failed to improve the clinical utility of existing simple and inexpensive scoring systems [6,7,8,9,10]. Combinations of markers and/or scoring systems potentially add value but lack external and/or multicenter validation [11,12,13,14]. The development and increasing accessibility of omics platforms have provided opportunities for prognostication based on genetic [15], transcriptomic [16], proteomic [17,18,19,20], metabolic profiling [21,22], and multi-platform omics analyses [23] Nevertheless, the application of these platforms in AP remains in its infancy.

The premise of machine learning (ML) in disease prognostication is to incorporate the wisdom embedded within decisions made by multiple clinicians, and the outcomes of their patients, in order to inform the individualized patient treatment [24]. ML is a broad field involving computer science and statistics, and broadly speaking, it involves a machine-led selection of iterative computational models to progressively improve the model’s performance in a specific task. The ability to handle vast datasets in an inherently unbiased manner has led to the growing interest in, and use of, ML-based applications in multiple areas of medicine [25,26,27,28]. This includes the use of ML in the diagnosis, prognosis, and predicted treatment response in patients with gastrointestinal diseases, although the lack of high-quality datasets continues to present a problem [29].

In AP, ML has been used to aid in the prediction of OF [30,31,32] and severity [33,34,35]; however, thus far, no study has accurately and timeously predicted the need for therapeutic intervention [36]. The identification of high-risk patients who require specialist intervention is critical, as these patients are, not only, at considerable risk of adverse disease outcomes but timely management has considerable implications for the health-care system. This includes the possible need to provide services that may not be available throughout the day, or every day in the week, and the provision of services may mean a transfer to a different hospital in some care settings.

Although there have been attempts to standardize the language surrounding indications for intervention in AP [37], there are numerous instances (e.g., ongoing OF or other severe gastrointestinal symptoms, due to the mass effect of walled-off necrosis or disconnected pancreatic duct syndrome) [38] that warrant intervention under the care of an experienced pancreatologist. These can often be difficult to classify or use to provide general guidance on the use of traditional methods.

Therefore, this study aimed to apply an ML algorithm to preoperatively predict the need for intervention and mortality in patients admitted with AP.

## 2. Materials and Methods

### 2.1. Overview

Data on patients with AP were collected, retrospectively (single center, Chengdu) and prospectively (two centers, Liverpool and Chengdu), and analyzed following the STROBE guidelines for observational studies [39]. Confirmation that specific ethical approval was not required was provided by the Institutional Review Board of West China Hospital of Sichuan University (WCH/SCU), due to prior approval for the use of retrospective data. Informed consent was obtained from patients admitted to Royal Liverpool University Hospital (RLUH), and ethical approval was not required because anonymized data were used. Predictors and outcomes from both retrospective and prospective databases were used to develop and test predictive models for intervention and mortality.

### 2.2. Cohorts and Data Collection

Eligible patients were identified from the Hospital Information System by using the International Classification of Diseases, 10th edition, code K85. All patients were admitted to WCH/SCU or RLUH, within 48 h of abdominal pain onset, with a diagnosis of AP, as defined by the revised Atlanta classification [37]. Patients in the retrospective cohort were admitted between 1 October 2009 and 30 September 2013. Patients in the prospective cohorts were admitted between 1 September 2014 and 31 December 2015 (WCH/SCU) or between 1 June 2010 and 30 June 2017 (RLUH). Data collection in both centers was based on a predefined pro forma and coordinated by experienced researchers, with quality assurance and control measures in place at every step of the study process.

### 2.3. Potential Predictors

Demographic variables (age, sex, comorbidities, abdominal pain onset time, and etiology), available quantitative laboratory tests on admission common to all three cohorts (white blood cell [WBC], neutrophils, lymphocytes, hematocrit, urea, creatinine, albumin, and C-reactive protein [CRP]), and clinical severity scores on admission (sequential organ failure assessment [SOFA] [40], systemic inflammatory response syndrome [SIRS] [41], bedside index, for severity in acute pancreatitis [BISAP] [42], acute physiology, and chronic health examination [APACHE] II [43], as well as modified computerized tomographic severity index [MCTSI]) [44] were collected. Additional clinical variables, including pleural effusion, local complications, OF, pancreatic, and extrapancreatic infection (bacteremia and others) were also recorded (worst during hospitalization or before surgery), as were daily assessments of type, onset, and duration of OF.

### 2.4. Definition of Groups

The patients were divided into conservative-treatment (no intervention) and invasive-intervention (including pancreatic cyst percutaneous catheter drainage and necrosectomy) groups for further analysis.

### 2.5. Statistical Analysis and Model Development

The chi-squared test was used to analyze categorical data, and the Kruskal–Wallis test was used for ranked variables. The rank-sum test was used for skewed and continuous data. Random forest (RF) ML [45] multivariate analysis was used to construct the algorithms and resolve the impact of data imbalances on predictions (2714 cases in the non-intervention group, more than 20 times of the 132 cases in the intervention group). RF can process high-dimensional samples and does not require dimensionality reduction for datasets with numerous variables. It is worth noting that RF is an ensemble method that utilizes many classifiers to work together, and it has high accuracy and superiority on unbalanced datasets. The mean decrease in the Gini value of each variable, indicating the importance of the variable to the outcome, was obtained by the varImpPlot function using the R software. We comprehensively evaluated the model’s performance, using the area under receiver-operating-characteristic curve (AUC) analysis, and evaluated the post-test probabilities by calculating the positive and negative likelihood ratios.

All the analytic processes were performed using R software (version 3.6.3).

#### 2.5.1. Data Sources

Since there were three datasets in this study: (1) a retrospective cohort from WCH/SCU, (2) a prospective cohort from WCH/SCU, (3) and a prospective cohort from RLUH, the differences between various data collection times and populations might have had varying effects on outcomes. Therefore, the differences affecting the research outcomes were analyzed. First, we used the three datasets, separately, to predict intervention needs and mortality. Thereafter, we aggregated the three datasets into a single dataset for the prediction. We found that the results of modelling the three datasets separately, and those of integrating them into one dataset, were similar. In addition, this study was a retrospective analysis of data collected in a previous period. Therefore, we consolidated the three different data sources into a single dataset before analysis and modeling.

#### 2.5.2. Univariate Analysis

The impact of each individual variable on “need for intervention” and “mortality” was examined using univariate analysis. Where the resulting *p*-value was <0.10, the variable was included in multivariable analysis.

#### 2.5.3. Performance of the ML Algorithm

**Figure 1:**
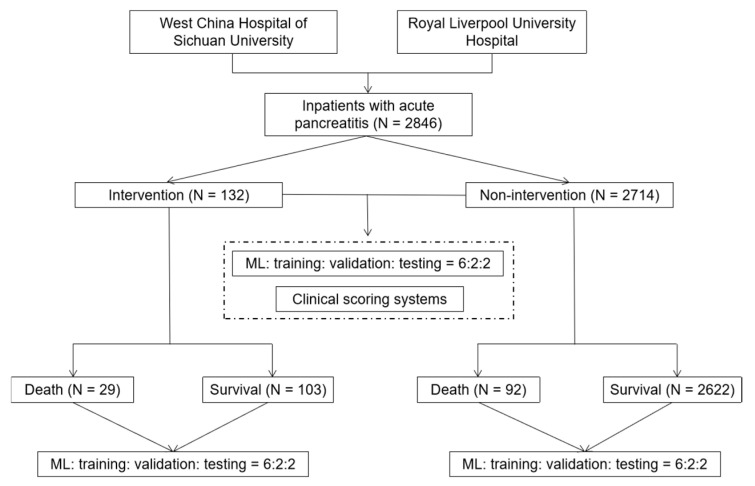
The flow chart of this study.

For multivariate analysis, an RF ML approach was used. Patients were divided into three groups for modeling: (1) intervention and conservative management, (2) mortality and survival among intervention patients, and (3) mortality and survival among conservatively managed patients. The larger the mean decrease in the Gini value, the greater the impact of the variable. We extracted the characteristics of the intervention and deceased patients, compared with those of non-intervention and surviving patients, and evaluated the model’s performance using evaluation indicators (accuracy, AUC, sensitivity, specificity, and likelihood ratio). Accuracy was evaluated based on the percentage of correct predictions. To predict the performance of ML, accuracy was evaluated based on the proportion of correct predictions in the total sample. As a rule of thumb, a test with a high predictive value has a positive likelihood ratio >5, usually closer to 10, and occasionally higher [46]. In all three groups, the total dataset was divided into training, validation, and test datasets according to a specific ratio of 6:2:2. The training set was used to develop the model, the validation set was used to adjust the parameters, and the test set was used to obtain the final result, which was the average performance with 30 repetitions (Figure 1). The hyperparameters of random forest include the number of trees (ntree), the number of variables required to build a single tree (nvariable), and the minimum sample size of leaf nodes (nodesize). Through parameter sensitivity analysis (Supplementary Table S1), the final chosen hyperparameters were: ntree = 500, nvariable = 4, and nodesize = 1. 

## 3. Results

### 3.1. Comparison of Characteristics between Intervention and Non-Intervention Patients with AP

A retrospective cohort of 2018 patients (WCH/SCU) and two prospective cohorts of 259 and 569 patients (WCH/SCU and RLUH, respectively) were included in the analysis. The proportions of intervention and mortality (chi-squared test; *p* = 0.432 and *p* = 0.411, respectively) were similar across all cohorts, indicating that any observed differences in the number of interventions and/or mortality were unlikely to be due to inherent differences in the source data. 

The clinical characteristics of the 2846 patients are summarized in Table 1. The number of patients requiring therapeutic intervention was 132 (4.6%), while 2714 (95.4%) were managed conservatively. The most common etiologies (in order) were biliary, hypertriglyceridemia, and alcohol consumption. The median age of all participants was 46 years (interquartile range, 38–58 years), and 64.0% were men. There were no significant differences in age, sex, Charlson comorbidity index, or etiology between the two groups. The time from pain to admission was 6 h longer in the intervention group (*p* < 0.05). 

WBC, neutrophil, hematocrit, urea, creatinine, and CRP in the intervention group were significantly higher than those in the non-intervention group, while albumin levels were lower (all *p* < 0.05). The admission clinical scoring systems, including SOFA, BISAP, SIRS, APACHE II, and worst MCTSI, were all higher among intervention patients, with the ratio of severe cases being three times higher than that in the non-intervention group.

Patients requiring intervention exhibited significantly worse clinical outcomes: 98/132 (74.2%) developed acute peripancreatic fluid collection, and 84/132 (63.6%) developed pancreatic and/or peripancreatic necrosis. Out of the 84 patients with necrosis, 81 were confirmed to have infectious necrosis; 99/132 (75%), 42/132 (31.8%), and 29/132 (22%) therapeutic-intervention patients developed persistent pulmonary, circulatory, and renal failure, respectively, with the duration of all three types’ OF lasting longer than those in the non-intervention group. Extrapancreatic infection was also more prevalent in the intervention group, regardless of bacteremia or lung infection.

The comparisons between death and survival among intervention patients, as well as among non-intervention patients, are shown in Supplementary Table S2 and Supplementary Table S3, respectively.

### 3.2. Important Features and Predictors for Intervention and Mortality

As shown in Table 2, important features (variables) associated with intervention and death differed. Compared with that in non-intervention patients, the duration of pulmonary failure was the most important factor in intervention patients. The remaining nine important variables for intervention patients, ranging from heavy to light, were neutrophils, albumin, lymphocytes, creatinine, age, hematocrit, onset of circulatory failure, APACHE II, and duration of circulatory failure. OF characteristics were all important variables for death among both intervention and non-intervention patients, especially for the occurrence of circulatory and renal failure. Circulatory failure, onset of circulatory failure, duration of circulatory failure, renal failure, duration of renal failure, duration of pulmonary failure, and APACHE II were all important variables for death in both intervention and non-intervention groups. The difference was that urea and CRP were important indicators of death in intervention patients, while creatine and WBC were important indicators in non-intervention patients.

**Figure 2:**
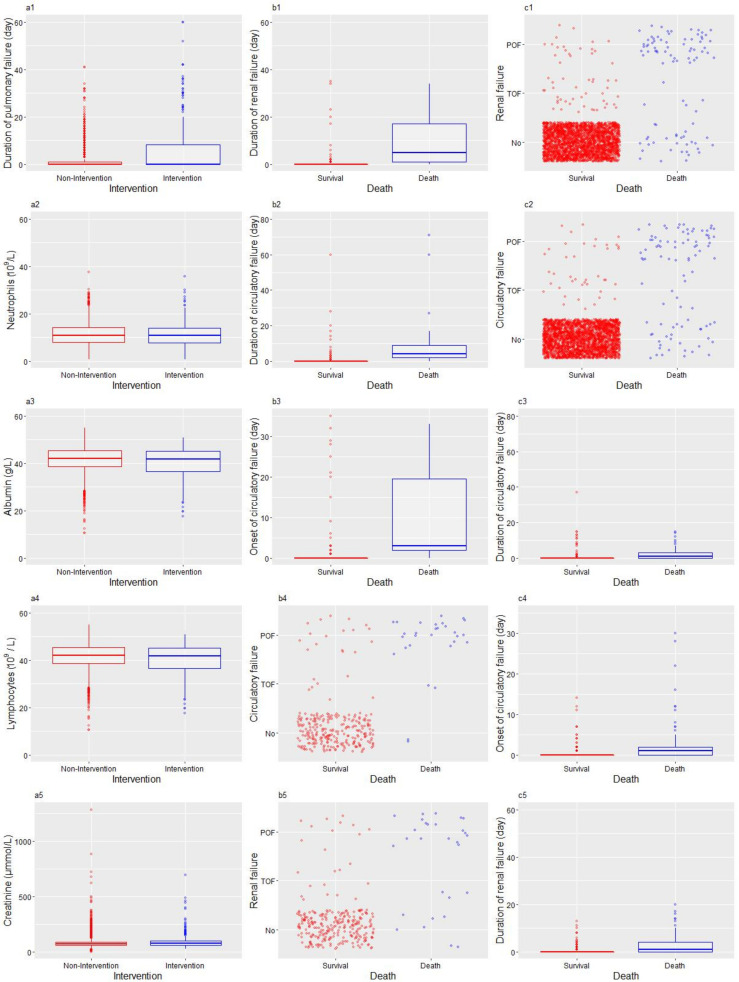
The relationship between important variable (the top five) and outcome. The first column (**a1**–**a5**) displays the top features for intervention, the second column (**b1**–**b5**) is for death in the intervention group, and the third column (**c1**–**c5**) is for death in the non-intervention group.

Figure 2 shows the relationship between important variables (the top five) and the outcome. The first column (a) displays the top features for intervention, the second column (b) is for death in the intervention group, and the third column (c) is for death in the non-intervention group. A scatter plot was used to show the relationship between categorical variables and the outcome, and a box plot was used to show the relationship between quantitative data and the outcome. Pulmonary failure persisted significantly longer in the intervention groups than in the non-intervention groups, along with higher neutrophil and creatinine levels and a lower albumin level, while the lymphocyte level was similar between these two groups. The top five important features of death were all about circulatory and renal failure. The difference between the intervention and non-intervention groups, among deceased patients, was that the duration of renal and circulatory failure had an impact on death in the intervention group, while the most important variables for death in the non-intervention group were the rate of renal failure and circulatory failure.

### 3.3. Prediction and Diagnostic Performance for Intervention and Mortality

Regarding the prediction of intervention, the accuracy of ML-based intervention prediction was 96%, thus indicating that predicting both the positive and negative categories of the model was highly accurate. The model identified 74% (sensitivity) of patients requiring intervention. Overall, the AUC was approximately 90%, and the positive likelihood ratio was 22.3. The death in the intervention patients were 86% recognized (sensitivity), the AUC reached 89%, and the positive likelihood ratio was 6.14. In terms of death in non-intervention prediction, the ML-based model performed better, the AUC could reach 98%, and the positive likelihood ratio was 69.6 (Table 3). The performance of all three ML models on the test dataset was consistent with the above-mentioned.

### 3.4. Comparison of the Models with Prognostic Scores

Furthermore, the predictive performance for intervention and mortality, in patients with AP, from the test set was compared among ML models, SOFA, BISAP, SIRS, APACHE II, and worst MCTSI by calculating the positive likelihood ratios and post-test probabilities. In the test set, 4.64% of patients with AP required intervention. The existing prognostic scores on admission showed minimal to small changes, with an increase in the likelihood of intervention in patients with AP with extremely low sensitivities, while only the ML model moderately increased the rate, with a positive likelihood ratio of 25.5 and post-test probability of 55.4%. On predicting mortality in all intervention patients, the ML model performed better, or equally, with prognostic scores. Interestingly, the ML model significantly improved the likelihood ratio (71.9) in predicting mortality in non-intervention patients, increasing the 3.39% pre-test probability to 71.6% (post-test probability), while the worst MCTSI showed nearly no change. The details are presented in Table 4.

## 4. Discussion

To the best of our knowledge, this is the first study to use ML to quantify AP intervention indications and predictors of mortality on admission. Based on substantial international AP data from two centers, we found the duration of pulmonary failure to be an important indicator of intervention, followed by neutrophils and albumin, and OF characteristics were important predictors of death, in patients with AP, by ML. Using our models, we can predict whether patients with AP require intervention at an early stage of hospitalization, thus providing an important reference for timely consideration of whether to transfer to the intervention department or a higher-level hospital that can perform intervention. Furthermore, a pre-judgment can also be made regarding death, especially in those non-intervention patients with AP. 

The use of big data to capture patient-level outcomes has increased exponentially over the past 10 years, providing a strong foundation for continuing investigations on questions more specific to surgery [47]. ML algorithms, based on big data from multiple sources, are being developed to help deliver care, inform health policy, and reduce waste, since various data sources can potentially yield a rich matched data set [48,49]. ML applications can improve the accuracy of treatment protocols and health outcomes through algorithmic processes [50]. While guidelines present evidence-based international consensus statements on AP management, mainly through the collaboration of a panel of experts, new and more instructive guidelines require more data to be implemented in this era of big data. 

Clinicians worldwide seem to be following the same initial, guideline-based management protocol to the greatest extent possible; nonetheless, surgeons hold different opinions regarding multidisciplinary strategies for endoscopy, radiology, and interventions. Most guidelines and related randomized controlled trials compared intervention methods [51,52,53,54,55,56] or timing [57] of interventions but investigated indicators minimally. In addition, although IPN is the intervention recommended by most AP treatment guides for necrotizing pancreatitis [38,58,59,60], it is often determined when the intervention approaches in clinical practice. Clinical indicators for predicting interventions on admission, using real-world big data, can balance clinical efficacy with cost effectiveness. To identify intervention patients in the early stage of hospitalization, we intended to use the data obtained on admission, as well as the worst preoperative imaging manifestations and OF characteristics, to identify predictors of intervention.

A prediction model was ultimately established. The better the predictive performance, the higher the accuracy of predicting whether a new patient with AP will be operated on or die. There were no existing prognostic scores for intervention in patients with AP, as our results demonstrated that the existing available AP-related prognostic systems showed low predictive performance for intervention. Our results revealed that the AUC for the prediction of intervention was not low, the intervention patient-recognition rate (sensitivity) was 74%, and patients who did not require surgery had recognition rates (specificity) exceeding 90%, suggesting that the model is useful for the initial screening of interventions that do not require surgery. Patients with AP who do not require intervention are ruled out first (because of high accuracy and specificity), and the remaining patients can be further observed to determine whether intervention is warranted, thus saving medical resources. Moreover, a positive likelihood ratio >5 indicated our model’s good predictive effect, while other prognostic scores at the early stage of the disease almost lacked predictive value in predicting interventions in patients with AP. 

The predictive performance for mortality was better with an AUC > 95% and a positive likelihood ratio > 10. This suggests that the model can be used to predict death in both interventions, more so in non-intervention patients, and attention can be focused on advancement. Regarding the top 10 variables important for death, whether the patient is operated on or not, the important variable was organ function, differing greatly from the variables important to intervention, and the other two studies predicted hospital mortality in patients with AP (Supplementary Table S4). The Dutch Pancreatitis Study Group concluded that infection, onset, and duration of OF were not associated with death in necrotizing pancreatitis [61], findings that are inconsistent with ours [62,63,64]. This may be because of the single center and multicenter analyses differed in their results. Therefore, we used a two-center study to further confirm that OF was more important than infection as a predictor of death in AP, based extensive AP data. 

Our study also has some limitations. Firstly, most of the data were collected on admission; however, the condition of the patients with AP changed over time. To predict surgery and death more accurately, more time-consuming variables or more frequent data collection are required for predictive research. Secondly, if invasive intervention was required, we usually performed selective percutaneous catheter drainage (pancreatic necrosis less than 30%) or a retroperitoneal pancreatic necrosectomy approach (pancreatic necrosis greater than 30%), but we did not perform percutaneous or endoscopic transgastric drainage routinely [62]. Comparison between open and minimally invasive procedures would modify the current model and require further analysis. Thirdly, the retrospective collection of data may not contain all the features needed for current or future studies, which makes it impossible to guarantee homogeneity between the local data and study data in model reproduction. Therefore, more prospective data sources in multi-regional and multi-center studies may strengthen the interpretation of model validation methods and, consequently, establish general models that can be widely promoted.

## 5. Conclusions

ML models are potentially useful in predicting intervention and death, in patients with AP, using clinical indicators on admission.

## Figures and Tables

**Table 1:** Characteristics between intervention and non-intervention patients with AP.

Characteristic	Total (*n* = 2846)	Intervention (*n* = 132)	Non-Intervention (*n* = 2714)	*p*
**Demographics**				
Age, year (M[Q])	46 (38–58)	48 (39–62)	46 (38–57)	0.125
Male (%)	1822 (64.0)	88 (66.7)	1734 (63.9)	0.578
CCI (M[Q])	0 (0–1)	0 (0–1)	0 (0–1)	0.260
Modified CCI, (M[Q])	0 (0–1)	0 (0–2)	0 (0–1)	0.176
ASA (%)				0.005
I	2120 (74.5)	108 (81.8)	2012 (74.1)	
II	573 (20.1)	13 (9.8)	560 (20.6)	
III	153 (5.4)	11 (9.3)	142 (5.2)	
From onset to admission, h (M[Q])	18 (10–27)	24 (10–33)	18 (10–27)	0.001
Aetiology (%)				0.063
Biliary	1069 (37.6)	65 (49.2)	1004 (37.0)	
Hypertriglyceridemia	805 (28.3)	33 (25.0)	772 (28.4)	
Alcoholics	216 (7.6)	8 (6.1)	208 (7.7)	
ERCP	20 (0.7)	0 (0.0)	20 (0.7)	
Drug-induced	8 (0.3)	1 (0.8)	7 (0.3)	
Others	728 (25.6)	25 (18.9)	703 (25.9)	
**Laboratory tests**				
WBC, 10^9^/L (M[Q])	12.9 (10.01–16.30)	14.3 (10.43–17.35)	12.87 (10–16.26)	0.011
Neutrophils, 10^9^/L (M[Q])	11.00 (8.10–14.34)	12.66 (9.17–15.61)	10.95 (8.05–14.28)	0.001
Lymphocyte, 10^9^/L (M[Q])	1.01 (0.70–1.49)	0.96 (0.62–1.53)	1.02 (0.70–1.49)	0.352
Hematocrit, % (M[Q])	43 (39–46)	45 (40–49)	43 (39.3–46)	0.003
Urea, mmol/L (M[Q])	5.00 (3.72–6.60)	6.36 (4.79–8.61)	4.92 (3.70–6.47)	<0.001 *
Creatinine, μmmol/L (M[Q])	74 (62–89)	87 (68–134)	73 (62–88)	<0.001 *
Albumin, g/L (M[Q])	42.0 (38.2–45.3)	37.3 (32.3–43.2)	42.1 (38.6–45.4)	<0.001 *
CRP, mg/L (M[Q])	28.7 (3.31–142)	158 (20–22)	26 (2.7–136)	<0.001 *
**Clinical scoring systems**				
SOFA (M[Q])	0 (0–2)	2 (0–3)	0 (0–1)	<0.001 *
BISAP (M[Q])	1 (0–2)	2 (1–2)	1 (0–2)	<0.001 *
SIRS (M[Q])	1 (1–2)	2 (1–3)	1 (1–2)	<0.001 *
APACHE II (M[Q])	4 (2–7)	7 (4–11)	4 (2–7)	<0.001 *
RAC (%)				<0.001 *
Mild	1373 (48.2)	4 (3.0)	1369 (50.4)	
Moderately severe	888 (31.2)	29 (22.0)	859 (31.7)	
Severe	585 (20.6)	99 (75.0)	486 (17.9)	
Worst MCTSI (M[Q])	2 (0–6)	8 (6–10)	2 (0–6)	<0.001 *
From admission to worst MCTSI, day (M[Q])	0 (0–2)	2 (1–9)	0 (0–1)	<0.001 *
**Clinical outcomes**				
*Local complication*				
APFC (%)	1121 (39.4)	98 (74.2)	1023 (37.7)	<0.001 *
Necrosis (%)	416 (14.6)	84 (63.6)	332 (12.2)	<0.001 *
*Single organ failure*				
Pulmonary failure (%)				<0.001 *
TOF	417 (14.7)	8 (6.1)	409 (15.1)	
POF	578 (20.3)	99 (75.0)	479 (17.6)	
Onset of pulmonary failure, day (M[Q])	0 (0–1)	1 (1–2)	0 (0–1)	<0.001 *
Duration of pulmonary failure, day (M[Q])	0 (0–1)	12.5 (1–24)	0 (0–1)	<0.001 *
Circulatory failure (%)				<0.001 *
TOF	42 (1.5)	9 (6.8)	33 (1.2)	
POF	111 (3.9)	42 (31.8)	69 (2.5)	
Onset of circulatory failure, day (M[Q])	0 (0–0)	0 (0–3)	0 (0–0)	<0.001 *
Duration of circulatory failure, day (M[Q])	0 (0–0)	0 (0–3)	0 (0–0)	<0.001 *
Renal failure (%)				<0.001 *
TOF	57 (2.0)	15 (11.4)	42 (1.5)	
POF	104 (3.7)	29 (22.0)	75 (2.8)	
Onset of renal failure, day (M[Q])	0 (0–0)	0 (0–1)	0 (0–0)	<0.001 *
Duration of renal failure, day (M[Q])	0 (0–0)	0 (0–1)	0 (0–0)	<0.001 *
*Pleural effusion* (%)	268 (9.4)	15 (11.4)	253 (9.3)	0.528
*IPN* (%)	85 (3.0)	81 (61.4)	4 (0.1)	<0.001 *
*Extrapancreatic infection* (%)				<0.001 *
Bacteremia	75 (2.6)	24 (18.2)	51 (1.9)	
Lung and others	147 (5.2)	31 (23.5)	116 (4.3)	

AP, acute pancreatitis; CCI, Charlson comorbidity index; ASA, American society of anesthesiologists; ERCP, endoscopic retrograde cholangiopancreatography; WBC, white blood cell count; CRP, C-reactive protein; SOFA, sequential organ failure assessment; BISAP, bedside index of severity in acute pancreatitis; SIRS, systemic inflammatory response syndrome; APACHE II, acute physiology and chronic health evaluation II; RAC, revised Atlanta classification; MCTSI, modified computerized tomographic severity index; APFC, acute peripancreatic fluid collection; IPN, infected pancreatic necrosis; TOF, transient organ failure; POF, persistent organ failure; M[Q], median and inter-quartile range for quantitative data; (%), number and percentage for categorical variables; * *p* < 0.05, indicates statistical significance.

**Table 2:** Top 10 important features for intervention or mortality among the three groups.

Intervention		Death in Intervention		Death in Non-Intervention	
Variable	Mean Decrease Gini	Variable	Mean Decrease Gini	Variable	Mean Decrease Gini
Duration of pulmonary failure	23.78	Duration of renal failure	2.54	Renal failure	10.99
Neutrophils	10.18	Duration of circulatory failure	2.52	Circulatory failure	10.00
Albumin	9.91	Onset of circulatory failure	2.35	Duration of circulatory failure	8.62
Lymphocytes	9.06	Circulatory failure	2.21	Onset of circulatory failure	7.70
Creatine	8.36	Renal failure	1.60	Duration of renal failure	6.37
Age	8.27	Creatinine	1.59	Onset of renal failure	5.46
Hematocrit	8.09	Duration of pulmonary failure	1.38	APACHE II	4.72
Onset of circulatory failure	7.95	Urea	1.19	Duration of pulmonary failure	4.45
APACHE II	6.70	APACHE II	1.19	Creatinine	4.09
Duration of circulatory failure	5.48	CRP	0.92	WBC	3.80

APACHE II, acute physiology and chronic health evaluation II; CRP, C-reactive protein; WBC, white blood cell count.

**Table 3:** Performance of prediction for the three groups.

	Accuracy	AUC	Sensitivity	Specificity	Likelihood Ratio (+)	Likelihood Ratio (−)
**Predicting Intervention in AP (*n* = 2846)**						
Validation (*n* = 569)	0.96	0.90	0.74	0.97	22.3	0.27
Test (*n* = 569)	0.97	0.91	0.76	0.97	25.5	0.35
**Predicting Death in Intervention (*n* = 132)**						
Validation (*n* = 26)	0.84	0.89	0.74	0.86	6.14	0.30
Test (*n* = 26)	0.82	0.89	0.82	0.82	4.80	0.28
**Predicting Death in Non-Intervention (*n* = 2714)**						
Validation (*n* = 543)	0.98	0.98	0.76	0.99	69.6	0.25
Test (*n* = 543)	0.98	0.99	0.77	0.99	71.9	0.31

**Table 4:** Performance of prediction with ML models and clinical scoring systems in the test set.

	Sensitivity	Specificity	Likelihood Ratio (+)	Post-Test Probability (%)
Intervention (4.64% pre-test probability)				
ML model	0.76	0.97	25.5	55.4
SOFA	0.08	0.98	5.0	19.6
BISAP	0.08	0.98	4.3	17.3
SIRS	0.06	0.98	3.2	13.5
APACHE II	0.08	0.98	5.4	20.8
Worst MCTSI	0.13	0.99	12.7	38.2
Death in intervention (21.97% pre-test probability)				
ML model	0.82	0.82	4.8	57.5
SOFA	0.69	0.78	3.7	51.0
BISAP	0.52	0.96	4.4	55.3
SIRS	0.44	0.84	2.3	39.3
APACHE II	0.69	0.92	6.4	64.3
Worst MCTSI	0.48	0.69	2.0	36.0
Death in non-intervention (3.39% pre-test probability)				
ML model	0.77	0.99	71.9	71.6
SOFA	0.11	0.99	21.5	43.0
BISAP	0.14	0.99	32.5	53.3
SIRS	0.07	0.99	12.7	30.8
APACHE II	0.15	0.99	30.2	51.4
Worst MCTSI	0.03	0.96	1.0	3.4

ML, machine learning; SOFA, sequential organ failure assessment; BISAP, bedside index of severity in acute pancreatitis; SIRS, systemic inflammatory response syndrome; APACHE II, acute physiology and chronic health evaluation II; MCTSI, modified computerized tomographic severity index.

## Data Availability

Restrictions apply to the availability of data generated or analysed during this study to preserve patient confidentiality or because they were used under license. The corresponding author will on request detail the restrictions and any conditions under which access to some data may be provided.

## Supplementary Materials

The following supporting information can be downloaded at: https://www.mdpi.com/article/10.3390/jpm12040616/s1, Table S1. Parameter sensitivity analysis. Table S2. Characteristics between died and survived intervention patients with AP. Table S3. Characteristics between died and survived non-intervention patients with AP. Table S4. Characteristics of studies predicted hospital mortality in patients with AP [65,66].
