# Supplementary material for: Predicting the Need for Therapeutic Intervention and Mortality in Acute Pancreatitis: A Two-Center International Study Using Machine Learning
Source: J Pers Med. 2022 Apr 11;12(4):616. doi: 10.3390/jpm12040616 (PMC9031087; doi:10.3390/jpm12040616)
Supplement: Supplementary file 1 [file jpm-12-00616-s001.zip › jpm-1648066-supplementary.pdf]

Table S1. Parameter sensitivity analysis

Parameter	AUC	95%CI
**ntree**		
50	0.749	0.618-0.880
100	0.752	0.652-0.852
200	0.756	0.670-0.842
300	0.758	0.674-0.842
400	0.759	0.677-0.841
500	0.759	0.677-0.841
**nvariable**		
2	0.755	0.649-0.861
3	0.758	0.674-0.842
4	0.759	0.675-0.843
5	0.755	0.663-0.847
6	0.758	0.676-0.840
**nodesize**		
1	0.759	0.690-0.828
2	0.758	0.676-0.840
3	0.755	0.671-0.839
4	0.755	0.665-0.845
5	0.753	0.665-0.841
6	0.755	0.659-0.851
7	0.757	0.669-0.845
8	0.759	0.677-0.841
9	0.755	0.671-0.839

AUC, area under the receiver operating characteristic curve; CI, confidence interval.

Table S2. Characteristics between died and survived intervention patients with AP

Characteristic	Total (*N* = 132)	Death (*N* = 29)	Survival (*N* = 103)	*P*
**Demographics**				
Age, year (M[Q])	48 (39-62)	51 (43-64)	47 (39-62)	0.206
Male (%)	88 (66.7)	19 (65.5)	69 (67.0)	1
CCI (M[Q])	0 (0-1)	0 (0-1)	0 (0-1)	0.780
Modified CCI, (M[Q])	0 (0-2)	0 (0-1)	0 (0-2)	0.295
ASA (%)				0.949
I	108 (81.8)	24 (82.8)	84 (81.6)	
II	13 (9.8)	3 (10.3)	10 (9.7)	
III	11 (9.3)	2 (6.9)	9 (8.7)	
From onset to admission, h (M[Q])	24 (10-33)	26 (14-33)	24 (10-33)	0.532
Aetiology (%)				0.060
Biliary	65 (49.2)	13 (44.8)	52 (50.5)	
HTG	33 (25.0)	5 (17.2)	28 (27.2)	
Alcoholics	8 (6.1)	5 (17.2)	3 (2.9)	
ERCP	0 (0.0)	0 (0.0)	0 (0.0)	
Drug-induced	1 (0.8)	0 (0.0)	1 (1.0)	
Others	25 (18.9)	6 (20.7)	19 (18.4)	
**Laboratory tests**				
WBC, 10^9/L (M[Q])	14.3 (10.43-17.35)	15.08 (11.74-18.40)	13.93 (10.21-16.98)	0.311
Neutrophils, 10^9/L (M[Q])	12.66 (9.17-15.61)	12.97 (10.51-16.78)	12.4 (8.88-15.12)	0.283
Lymphocyte, 10^9/L (M[Q])	0.96 (0.62-1.53)	1.33 (0.79-1.65)	0.92 (0.60-1.47)	0.070
Hematocrit, % (M[Q])	45 (40-49)	46 (41-53)	45 (39-48)	0.116
Urea, mmol/L (M[Q])	6.36 (4.79-8.61)	9.10 (5.71-12.43)	5.96 (4.59-8.12)	0.004*
Creatinine, μmmol/L (M[Q])	87 (68-134)	150 (98-221.3)	81.3 (64.9-106)	<0.001*
Albumin, g/L (M[Q])	37.3 (32.3-43.2)	35.6 (31.7-39.2)	39 (32.7-44)	0.035
CRP, mg/L (M[Q])	158 (20-22)	208.5 (145.5-254)	135 (8.5-210)	0.002*
**Clinical scoring systems**				
SOFA (M[Q])	2 (0-3)	3 (1-6)	2 (0-3)	0.003*
BISAP (M[Q])	2 (1-2)	2 (2-3)	2 (1-2)	<0.001*
SIRS (M[Q])	2 (1-3)	2 (2-3)	2 (1-3)	0.008*
APACHE II (M[Q])	7 (4-11)	10 (8-16)	6 (3-9)	<0.001*
RAC (%)				0.002*
Mild	4 (3.0)	0 (0.0)	4 (3.9)	
Moderately severe	29 (22.0)	0 (0.0)	29 (28.2)	
Severe	99 (75.0)	29 (100.0)	70 (68)	
Worst MCTSI (M[Q])	8 (6-10)	8 (6-10)	8 (6-9)	0.384
From admission to worst MCTSI, day (M[Q])	2 (1-9)	2 (1-6)	2 (1-10.5)	0.358
**Clinical outcomes**				
Local complication				
APFC (%)	98 (74.2)	25 (86.2)	73 (70.9)	0.153
Necrosis (%)	84 (63.6)	19 (65.5)	65 (63.1)	0.984
Single organ failure				

Pulmonary failure (%)				0.002*
TOF	8 (6.1)	0 (0.0)	8 (7.8)	
POF	99 (75.0)	29 (100.0)	70 (68.0)	
Onset of pulmonary failure, day (M[Q])	1 (1-2)	1 (1-2)	1 (1-2)	0.458
Duration of pulmonary failure, day (M[Q])	12.5 (1-24)	24 (16-31)	8 (1-21)	<0.001*
Circulatory failure (%)				<0.001*
TOF	9 (6.8)	2 (6.9)	7 (6.8)	
POF	42 (31.8)	25 (86.2)	17 (16.5)	
Onset of circulatory failure, day (M[Q])	0 (0-0)	3 (2-21)	0 (0-0)	<0.001*
Duration of circulatory failure, day (M[Q])	0 (0-0)	4 (2-9)	0 (0-0)	<0.001*
Renal failure (%)				<0.001*
TOF	15 (11.4)	3 (10.3)	12 (11.7)	
POF	29 (22.0)	19 (65.5)	10 (9.7)	
Onset of renal failure, day (M[Q])	0 (0-0)	1 (1-2)	0 (0-0)	<0.001*
Duration of renal failure, day (M[Q])	0 (0-0)	5 (1-17)	0 (0-0)	<0.001*
Pleural effusion (%)	15 (9.4)	1 (3.4)	14 (13.6)	0.234
IPN (%)	81 (61.4)	19 (65.5)	62 (60.2)	0.761
Extrapancreatic infection (%)				0.719
Bacteraemia	24 (18.2)	4 (13.8)	20 (19.4)	
Lung and others	31 (23.5)	8 (27.6)	23 (22.3)	

AP, acute pancreatitis; CCI, Charlson comorbidity index; ASA, American society of anesthesiologists; ERCP, endoscopic retrograde cholangiopancreatography; WBC, white blood cell count; CRP, C-reactive protein; SOFA, sequential organ failure assessment; BISAP, bedside index of severity in acute pancreatitis; SIRS, systemic inflammatory response syndrome; APACHE II, acute physiology and chronic health evaluation II; RAC, revised Atlanta classification; MCTSI, modified computerized tomographic severity index; APFC, acute peripancreatic fluid collection; IPN, infected pancreatic necrosis; TOF, transient organ failure; POF, persistent organ failure; M[Q], median and inter-quartile range for quantitative data; (%), number and percentage for categorical variables; *$P<0.05$ indicates statistical significance.

Table S3. Characteristics between died and survived non-intervention patients with AP

Characteristic	Total (*N* = 2714)	Death (*N* = 92)	Survival (*N* = 2622)	*P*
**Demographics**				
Age, year (M[Q])	46 (38-57)	52.5 (43-67)	46 (38-57)	<0.001*
Male (%)	1734 (63.9)	56 (60.9)	1678 (64.0)	0.615
CCI (M[Q])	0 (0-1)	0 (0-2)	0 (0-1)	0.035*
Modified CCI, (M[Q])	0 (0-1)	0 (0-1)	0 (0-1)	0.938
ASA (%)				0.007*
I	2012 (74.1)	79 (85.9)	1933 (73.7)	
II	560 (20.6)	7 (7.6)	553 (21.1)	
III	142 (5.2)	6 (6.5)	136 (5.2)	
From onset to admission, h (M[Q])	18 (10-27)	24 (19-28)	17 (9-27)	<0.001*
Aetiology (%)				0.414
Biliary	1004 (37.0)	34 (37.0)	970 (37.0)	
HTG	772 (28.4)	34 (37.0)	738 (28.1)	
Alcoholics	208 (7.7)	6 (6.5)	202 (7.7)	
ERCP	20 (0.7)	0 (0.0)	20 (0.8)	
Drug-induced	7 (0.3)	0 (0.0)	7 (0.3)	
Others	703 (25.9)	18 (19.6)	685 (26.1)	
**Laboratory tests**				
WBC, 10^9/L (M[Q])	12.87 (10-16.26)	16.59 (12.68-20.8)	12.8 (9.97-16.1)	<0.001*
Neutrophils, 10^9/L (M[Q])	10.95 (8.05-14.28)	14.03 (10.33-17.15)	10.9 (8-14.12)	<0.001*
Lymphocyte, 10^9/L (M[Q])	1.02 (0.70-1.49)	1.16 (0.76-1.66)	1.01 (0.70-1.48)	0.133
Hematocrit, % (M[Q])	43 (39.3-46)	46.5 (42-51)	43 (39.1-46)	<0.001*
Urea, mmol/L (M[Q])	4.92 (3.70-6.47)	8.43 (5.92-12.03)	4.86 (3.65-6.34)	<0.001*
Creatinine, μmmol/L (M[Q])	73 (62-88)	144.5 (95-230.5)	73 (62-87)	<0.001*
Albumin, g/L (M[Q])	42.1 (38.6-45.4)	35.8 (28.9-39.6)	42.4 (38.9-45.5)	<0.001*
CRP, mg/L (M[Q])	26 (2.7-136)	171.3 (97.0-265.3)	23.4 (2-129)	<0.001*
**Clinical scoring systems**				
SOFA (M[Q])	0 (0-1)	3 (2-5)	0 (0-1)	<0.001*
BISAP (M[Q])	1 (0-2)	3 (2-4)	1 (0-2)	<0.001*
SIRS (M[Q])	1 (1-2)	3 (2-3)	1 (0-2)	<0.001*
APACHE II (M[Q])	4 (2-7)	12 (8-16.25)	4 (2-6)	<0.001*
RAC (%)				<0.001*
Mild	1369 (50.4)	0 (0.0)	1369 (52.2)	
Moderately severe	859 (31.7)	1 (1.1)	858 (32.7)	
Severe	486 (17.9)	91 (98.9)	395 (15.1)	
Worst MCTSI (M[Q])	2 (0-6)	0 (0-6)	2 (0-6)	0.967
From admission to worst MCTSI, day (M[Q])	0 (0-1)	0 (0-2)	0 (0-1)	0.194
**Clinical outcomes**				
Local complication				
APFC (%)	1023 (37.7)	68 (73.9)	955 (36.4)	<0.001*
Necrosis (%)	332 (12.2)	25 (27.2)	307 (11.7)	<0.001*
Single organ failure				
Pulmonary failure (%)				<0.001*

TOF	409 (15.1)	2 (2.2)	407 (15.5)	
POF	479 (17.6)	89 (96.7)	390 (14.9)	
Onset of pulmonary failure, day (M[Q])	0 (0-1)	1 (1-1.25)	0 (0-1)	<0.001*
Duration of pulmonary failure, day (M[Q])	0 (0-1)	4 (2-9)	0 (0-1)	<0.001*
Circulatory failure (%)				<0.001*
TOF	33 (1.2)	9 (9.8)	24 (0.9)	
POF	69 (2.5)	52 (56.5)	17 (0.6)	
Onset of circulatory failure, day (M[Q])	0 (0-0)	1 (0-2)	0 (0-0)	<0.001*
Duration of circulatory failure, day (M[Q])	0 (0-0)	1 (0-3)	0 (0-0)	<0.001*
Renal failure (%)				<0.001*
TOF	42 (1.5)	6 (6.5)	36 (1.4)	
POF	75 (2.8)	56 (60.9)	19 (0.7)	
Onset of renal failure, day (M[Q])	0 (0-0)	1 (0-2)	0 (0-0)	<0.001*
Duration of renal failure, day (M[Q])	0 (0-0)	1 (0-4)	0 (0-0)	<0.001*
Pleural effusion (%)	253 (9.3)	5 (5.4)	248 (9.5)	<0.001*
IPN (%)	4 (0.1)	0 (0.0)	4 (0.2)	1.000
Extrapancreatic infection (%)				<0.001*
Bacteraemia	51 (1.9)	10 (10.9)	41 (1.6)	
Lung and others	116 (4.3)	12 (13.0)	104 (4.0)	

AP, acute pancreatitis; CCI, Charlson comorbidity index; ASA, American society of anesthesiologists; ERCP, endoscopic retrograde cholangiopancreatography; WBC, white blood cell count; CRP, C-reactive protein; SOFA, sequential organ failure assessment; BISAP, bedside index of severity in acute pancreatitis; SIRS, systemic inflammatory response syndrome; APACHE II, acute physiology and chronic health evaluation II; RAC, revised Atlanta classification; MCTSI, modified computerized tomographic severity index; APFC, acute peripancreatic fluid collection; IPN, infected pancreatic necrosis; TOF, transient organ failure; POF, persistent organ failure; M[Q], median and inter-quartile range for quantitative data; (%), number and percentage for categorical variables; **P*<0.05 indicates statistical significance.

Table S4. Characteristics of studies predicted hospital mortality in patients with AP

Author and Year	Study design	Data collection time	Indicators	Sample size	AUROC	Sensitivity/ Specificity
Mofidi et al 2007 [65]	Prospective database	first presentation to hospital and at 48 hours.	Age; Hypotension unresponsive to fluid resuscitation; SIRS; Arterial PO2; LDH; Glucose; Urea; Ca^{2+}; Haematocrit; WBC	Training set: 398<br>Validation set: 100<br>Test set: 166	0.975	0.88/0.98
Ding et al 2021 [66]	MIMIC-III	within 24 hours after admission	Age; ALT; TB; CK-MB; PT; WBC; Amlyse; Ca2+; Creatine; Haematocrit; Lactate; Lipase	Training set: 269<br>Test set: 68	0.769	0.666/0.661

AP, acute pancreatitis; AUROC, area under the receiver operating characteristic curve; SIRS, systemic inflammatory response syndrome; LDH, lactate dehydrogenase; WBC, white blood cell; MIMIC-III, Medical Information Mart for Intensive Cart III; ALT, alanine aminotransferase; TB, total bilirubin; CK-MB, creatine kinase; PT, prothrombin time.
